# Structure and Magnetic Properties of Fe_1.95_P_0.8−*x*_Si_0.2_B*_x_* Compounds: Pushing Beyond the Orthorhombic Limit

**DOI:** 10.3390/ma19081579

**Published:** 2026-04-15

**Authors:** Bao Dorina, Lingling Bao, Borjigin Axida, Bao Wurentuya, Zhao Surilemu, Francois Guillou, Hargen Yibole

**Affiliations:** 1 College of Physics and Electronic Information, Inner Mongolia Key Laboratory of Applied Condensed Matter Physics, Inner Mongolia Normal University, 81 Zhaowuda Rd, Hohhot 010022, China; 2 Université Caen Normandie, ENSICAEN, CNRS, Normandie Univ, CRISMAT UMR6508, F-14000 Caen, France

**Keywords:** structure–property relationships, magnetic properties, magnetic anisotropy, magnetocaloric materials, permanent magnets

## Abstract

Fe_2_P compounds have recently attracted significant attention due to their large anisotropy and magnetization, making them promising candidates as hard magnetic materials. However, their relatively low Curie temperature limits practical applications. Previous studies have shown that substituting Si for P or Co for Fe increases the Curie temperature; however, Si substitution induces a hexagonal to orthorhombic structural transformation, while Co substitution reduces saturation magnetization. This work examines the evolution of the crystal structure and magnetic properties upon B substitutions in Fe_1.95_P_0.8−*x*_Si_0.2_B_*x*_ compounds close to the hexagonal/orthorhombic transformation. We show that B can increase the Curie temperature up to 675 K and the saturation magnetization to 139 A·m^2^·kg^−1^, while preserving the hexagonal structure beyond the limit allowed by Si substitutions only. X-ray diffraction of magnetically aligned powders confirms a uniaxial easy axis along the *c* axis and significant room-temperature magnetocrystalline anisotropy. The optimization of the intrinsic magnetic properties based on only metalloid substitutions paves the way for further development of this material family as rare-earth-free permanent magnets.

## 1. Introduction

For two decades, compounds derived from Fe_2_P have attracted interest, owing to their giant magnetocaloric effect. The original representative example is the (Mn,Fe)_2_(P,As) system, which exhibits a giant isothermal entropy change associated with a first-order ferromagnetic transition with limited hysteresis [1]. This discovery established the (Mn,Fe)_2_(P,*X*) family with *X* = As, Si, Ge, B as a key class of magnetocaloric materials. In particular, Si-based variants have progressively replaced the toxic As-containing or critical Ge-based analogs as the primary focus of research in this field [2,3]. More recently, the growing interest in developing rare-earth-free permanent magnets has renewed attention toward Fe_2_P-type compounds, not for their phase transition, but for their unusually large magnetocrystalline anisotropy for a transition-metal-based compound.

Hard magnetic materials are indispensable functional materials and their importance will further increase with the global transition toward cleaner energy technologies. Currently, the market is dominated in volume by low-performance yet cost-competitive ferrites (e.g., BaFe_12_O_19_ or SrFe_12_O_19_) and in value by rare-earth-based magnets (mainly Nd-Fe-B or Sm-Co alloys), which exhibit particularly high |*BH*|_max_ energy products. Concerns regarding the environmental impact and economic cost of rare earth elements have stimulated efforts to develop permanent magnets with reduced rare-earth content [4,5,6,7]. Although it remains challenging for rare-earth-free magnets to reach the performance of Nd–Fe–B or Sm–Co materials, one proposed strategy is the development of cost-competitive magnets with intermediate performance [7]. These “gap magnets” should be based on abundant raw materials, while also exceeding the performance of existing ferrite magnets. Several potential material families have been identified, some of which have attracted significant research interest, including Mn-based magnets such as MnAl and MnBi [8,9,10] and Co-based compounds such as Hf–Co and Zr–Co [11,12,13,14,15]. On the Fe-alloy side, most studies focus on α’’-Fe_16_N_2_ which offers great promise, in particular in terms of saturation magnetization, but its complex chemistry remains difficult to control [16,17,18]. Among other Fe-based materials, the Fe_2_P family stands out for its particularly large anisotropy [19]. However, the development of permanent magnets based on Fe_2_P is still at an early stage of research, due to compositions and preparation methods not yet fully optimized to achieve strong hard magnetic properties.

The Fe_2_P compound crystallizes in a hexagonal structure (space group $P\bar{6}2m)$ and contains two inequivalent metal sites, 3*f* (*x*_f_, 0, 0) and 3*g* (*x*_g_, 0, 1/2), located in tetrahedron and pyramidal environments of P atoms, respectively, forming distinctive layers stacked along the *c* axis. Binary Fe_2_P is ferromagnetic with high magnetization and an easy magnetization axis along *c* [20]. However, the Curie temperature of 214 K is too low for permanent magnet applications.

Substitution at the Fe sites with elements like Ni or Co, or at the P sites with elements such as As, Si, or B, can effectively increase the Curie temperature. However, metal site substitutions often result in a reduction in saturation magnetization [21], while substitutions on P face solubility limits or induce the formation of competing crystal structures [22].

Predicted by theory and demonstrated in a preliminary polycrystalline study, simultaneous Co for Fe and Si for P substitutions maintain high saturation magnetization and uniaxial magnetocrystalline anisotropy, while significantly enhancing *T*_C_ [23,24]. (Fe,Co)_2_(P,Si) single crystals have confirmed strong magnetocrystalline anisotropy for transition-metal compounds (*K*_1_ ≈ 0.9–1.1 MJ m^−3^ at room temperature) and a theoretical energy product of 165–204 kJ m^−3^ [25,26]. Submicrometric particles produced by ball milling have shown finite coercivities (*H*_C_ of the order of 2 kOe) in quaternary (Fe,Co)_2_(P,Si), ternary Fe_2_(P,Si) or quaternary (Fe,Co)_2_(P,Si or B) compounds [24,27,28,29,30]. Despite recent progress, several challenges remain to be addressed. In particular, the preparation method is critically important for observing hard magnetic properties, as is also the case for other rare-earth-free magnets [31]. Such investigations would be facilitated in Fe_2_P materials if carried out on already optimized compositions.

Si for P substitutions are particularly effective at increasing both the Curie temperature and the saturation magnetization. Unfortunately, the stability window of the hexagonal Fe_2_P-type phase at room temperature remains limited to Si < 0.25 [28]. In quaternary (Fe,Co)_2_(P,Si,B), Co for Fe substitutions have been shown to extend the stability of the hexagonal structure toward higher Si contents [24]; however, this approach comes at the cost of reduced saturation magnetization due to the smaller magnetic moment of Co compared with Fe [32,33].

An alternative strategy is explored in the present work: introducing B for P substitutions in a Fe_1.95_P_0.8−*x*_Si_0.2_B*_x_* composition deliberately selected at the upper Si limit of the hexagonal stability range, just before the hexagonal-to-orthorhombic structural transition occurs. The underlying hypothesis is that B substitution may enhance the Curie temperature without destabilizing the hexagonal structure or sacrificing magnetization of the Fe sites.

Previous studies involving B for P substitutions—whether in parent Fe_2_P, magnetocaloric MnFe(P,Si,B), or permanent-magnet candidate (Fe,Co)_2_(P,B)—have consistently reported an increase in Curie temperature [24,34,35]. However, these investigations were performed well within the hexagonal range and did not approach the orthorhombic boundary, where structural instability may critically influence magnetic properties. Therefore, a systematic study near this structural limit is required. In particular, understanding how B substitution affects the crystal structure, Curie temperature, magnetization, and the easy magnetic axis is essential for further developing Fe_2_(P,Si,B) compounds as rare-earth-free permanent magnets.

## 2. Materials and Methods

A series of Fe_1.95_P_0.8−*x*_Si_0.2_B*_x_* (*x* = 0, 0.025, 0.05, 0.075, 0.10, 0.125, 0.15, and 0.20) compounds were prepared by ball milling followed by a solid-state reaction. The elemental starting materials, Fe powder (>99.9%), P powder (>98.9%), Si lump (>99.999%), and B (>99.5%) pieces (Thermo Scientific Chemicals, Waltham, MA, USA), were ball-milled in a stainless-steel jar using a high-energy planetary mill (Pulverisette 4, Fritsch, Idar-Oberstein, Germany) at 360 rpm, using a sample-to-ball mass ratio of 1:5. The resulting powder was then compacted in a die with an inner diameter of 10 mm and uniaxially pressed into cylindrical pellets under a load of 8 T. The pellets were then sealed in quartz ampoules backfilled with 200 mbar of Ar. The heat treatment consisted of a single annealing step at 1100 °C for 24 h, followed by quenching in water. The annealed samples were manually ground into powders and sieved with a particle size of less than 36 µm for powder X-ray diffraction and magnetic alignment. For the preparation of magnetically aligned samples, the polycrystalline powder was mixed with epoxy resin in a 1:1 mass ratio to form a highly diluted mixture. This mixture was shaped into plates with approximate dimensions of 15 × 30 × 2 mm^3^, aligned under a magnetic field of μ_0_*H* = 1.1 T, with the surface of the plate being perpendicular to the magnetic field direction.

Powder X-ray diffraction (XRD) experiments were performed on a Empyrean diffractometer (PANalytical, Malvern, UK) using Cu-Kα radiation over a 2*θ* range of 20° to 90°. The Rietveld method, implemented in the FullProf software package (April 2023 version), was employed for structural refinement [36]. Magnetic measurements were carried out using the Quantum Design (San Diego, CA, USA) Versalab and PPMS systems equipped with a vibrating sample magnetometer option. For high-temperature magnetic measurements (*T* > 400 K), a Lakeshore (Carson, CA, USA) 7407 VSM with a high-temperature furnace attachment was used. Differential scanning calorimetry was conducted on a TA Instruments (New Castle, DE, USA) DSC 2500, equipped with a liquid nitrogen cooling pump and using standard aluminum crucibles.

A second batch of *x* = 0.05 and 0.10 samples was prepared to check the reproducibility. The characterization of these samples and its comparison with the original main batch can be found in the Supplementary Materials. This second batch of samples confirmed the main dataset, particularly for critically important properties such as Curie temperature and saturation magnetization, showing only a few percent (<3%) difference compared to the first batch.

## 3. Results and Discussion

**Figure 1:**
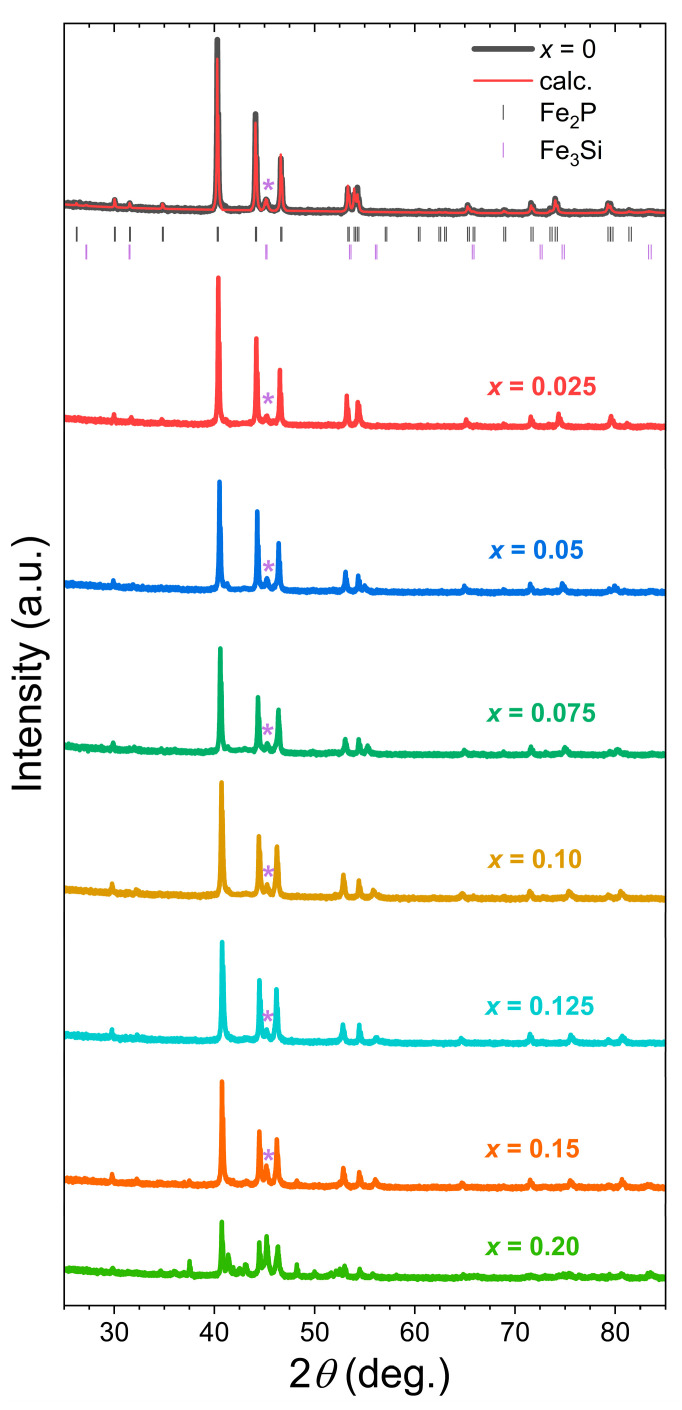
Room-temperature powder XRD patterns for Fe_1.95_P_0.8−*x*_Si_0.2_B*_x_* compounds. For *x* = 0, the experimental pattern is compared to Rietveld refinement and the below scatter points mark out the reflections for the main Fe_2_P-type (top) and secondary Fe_3_Si phases (bottom marks). The asterisk indicates the most intense and characteristic reflection of the Fe_3_Si phase. For clarity, for *x* > 0, only the experimental patterns were provided; the refinements can be found in the Supplementary Materials.

Figure 1 presents powder X-ray diffraction patterns for Fe_1.95_P_0.8−*x*_Si_0.2_B*_x_* compounds with *x* = 0, 0.025, 0.05, 0.075, 0.10, 0.125, 0.15, and 0.20 recorded at room temperature. The refinement results confirm that the boron-free sample, Fe_1.95_P_0.8_Si_0.2_, crystallizes in the hexagonal Fe_2_P-type structure (space group $P\bar{6}2m)$, with a limited amount of secondary phase (4.6 (1) *wt*% of Fe_3_Si, space group $FM\bar{3}M).$ The main Fe_2_P-type phase is stable up to *x* = 0.125, and the Fe_3_Si phase content remains approximately constant at 4–5 *wt*% across the range 0 ≤ *x* ≤ 0.125. A small amount of 3:1 phase is rather common in this material family when prepared by a solid-state reaction and is primarily ascribable to the facilitated formation of Fe_3_Si around 900 °C. While its content can be minimized by quenching and reducing the nominal metal:metalloid ratio [24], its presence remains difficult to eliminate completely or requires high-rate quenching methods (e.g., drop synthesis or melt spinning) [37]. It is worth noting that while the formation of Fe_3_Si can reduce the Curie temperature by depleting Si from the main phase, this effect is expected to be limited along our series since the Fe_3_Si content does not vary within the solid-solution range of *B* (0 ≤ *x* ≤ 0.125). Consequently, the observed evolution of the structure and properties can be primarily attributed to the boron substitutions themselves. Starting at *x* = 0.15 and most particularly noticeable at *x* = 0.20, one observes the appearance of additional diffraction peaks marking out the appearance of new phases and therefore the solubility limit for B in Fe_1.95_P_0.8−*x*_Si_0.2_B*_x_*. While a refinement could not be performed for *x* = 0.20, phase identification suggested the coexistence of hexagonal Fe_2_P, cubic Fe_3_Si, hexagonal Fe_5_Si_3_ and possibly BCO Fe_2_P phases. For *x* ≤ 0.125, upon increasing the B content, the (300) diffraction peak progressively shifts to lower angles (from 53.3° to ~52.8°), while the (002) peak shifts to higher angles (from 53.9° to ~56.2°), indicating a concurrent expansion of the *a* axis and the most pronounced contraction of the *c* axis.

**Figure 2:**
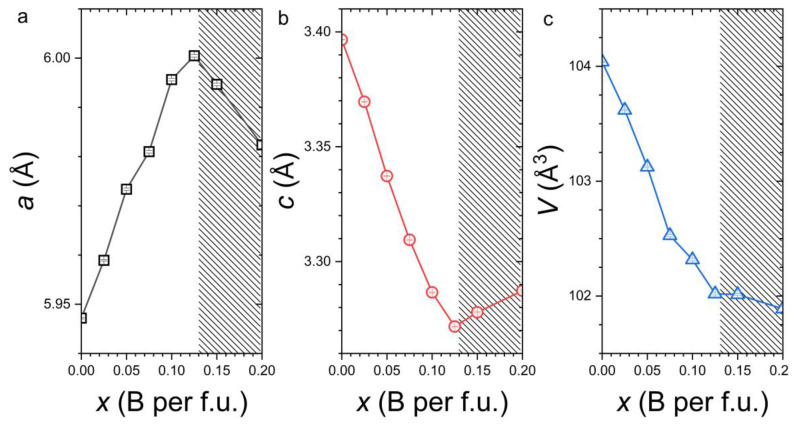
Evolution of the *a* (**a**) and *c* (**b**) cell parameters and unit cell volume *V* (**c**), for Fe_1.95_P_0.8−*x*_Si_0.2_B*_x_* compounds determined from XRD refinements. The stripe pattern marks out the composition above the solubility limit.

Figure 2 shows the unit cell volume and lattice parameters of the Fe_2_P-type main phase as determined by Rietveld refinement (presented in the Supplementary Materials). For the boron-free sample (*x* = 0), the refined lattice parameters differ slightly but remain within the range previously reported for off-stoichiometric Fe_1.95_P_0.8_Si_0.2_ [28].

Upon boron substitution, a progressive decrease in unit cell volume is observed, which directly reflects the smaller radius of B compared to P and indicates that B is effectively incorporated on the P sublattice. We note that while XRD is poorly sensitive to light elements, the possibility of B for P substitutions in the Fe_2_P-type structure was confirmed by neutron diffraction in MnFe(P,Si,B) magnetocaloric compounds [38]. The volume contraction nearly follows Vegard’s law and aligns with trends reported in related systems, including Fe_2_(P,B) ternary, MnFe(P,Si,B), and Fe_1.8_Co_0.2_(P,B) quaternary compounds [24,34,35]. Despite the significant differences in composition and lattice parameters among these host compounds, the boron solubility limit consistently lies around *x* ≈ 0.10–0.15. This convergence strongly suggests that the B solubility in Fe_2_P compounds is not primarily dictated by the host phase, but rather by Hume–Rothery constraints, namely the combined effects of atomic size mismatch and valence differences between B and P.

With increasing B content, a pronounced reduction in the *c/a* ratio is observed, decreasing from 0.571 to 0.545 as *x* increases from 0 to 0.125. This anisotropic lattice response indicates that boron substitution primarily affects the interlayer spacing along the *c* direction rather than producing an isotropic contraction of the lattice. Importantly, and in contrast to (Fe,Co)_2_(P,Si) compounds with Si > 0.2, this reduction in the *c/a* ratio can be achieved solely through metalloid substitution while preserving the hexagonal Fe_2_P structure. Such an isostructural evolution effectively brings the Fe 3*f* and 3*g* layers closer together.

It is particularly noteworthy that B substitution introduces a larger change in electron count than Si substitution while simultaneously driving the *c/a* ratio to lower values, yet no hexagonal-to-orthorhombic structural transition occurs. This observation indicates that electronic effects alone cannot account for the stabilization of the orthorhombic phase. Instead, it suggests that an average size factor plays a role in driving the structural instability toward the orthorhombic phase. This interpretation is consistent with the evolution observed in (Fe,Co)_2_(P,Si), where substitution of the smaller Co atom for Fe compensates the lattice expansion induced by Si and thereby stabilizes the hexagonal structure beyond the stability limit of the ternary Fe_2_(P,Si) system.

However, a purely size-based argument is insufficient to fully explain the structural behavior of Fe_2_P-derived systems. In the Fe_2_(P,As) series, isoelectronic substitution of As for P results in a significantly larger unit cell expansion than that observed in Fe_2_(P,Si), yet the orthorhombic phase still does not develop [39]. This comparison indicates that the orthorhombic distortion is not triggered solely by lattice expansion. Instead, the emergence of the orthorhombic phase most likely requires the simultaneous fulfillment of two structural conditions: a sufficiently large unit cell volume together with a strongly reduced *c/a* ratio. Conversely, if either of these conditions is not satisfied, the hexagonal Fe_2_P structure remains stable, as appears to be the case throughout the present Fe_1.95_P_0.8−*x*_Si_0.2_B*_x_* series with *x* < 0.15.

**Figure 3:**
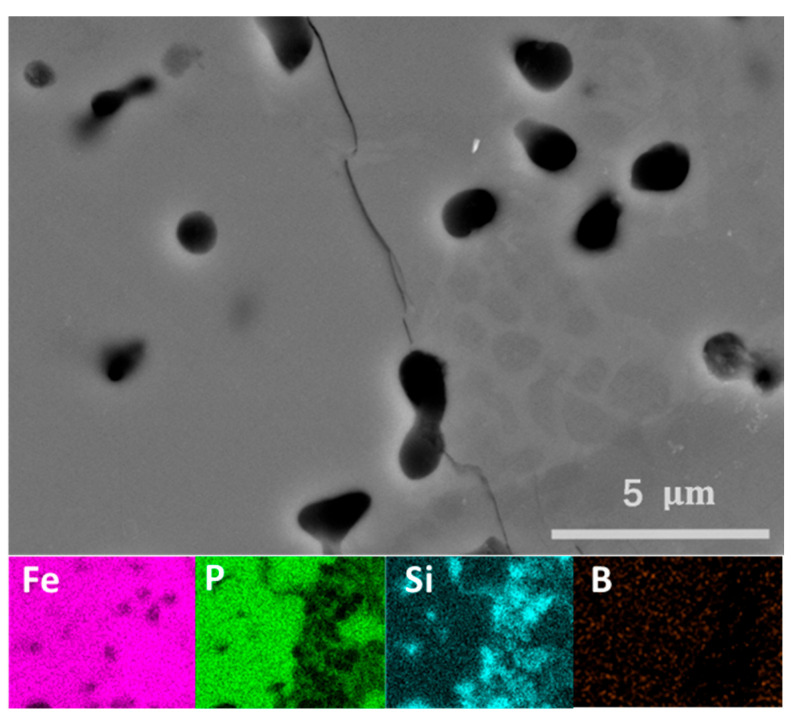
SEM (**top**) and EDX (**bottom**) images for Fe_1.95_P_0.75_Si_0.2_B_0.05_.

Electron microscopy and energy-dispersive X-ray analyses were carried out for several representative samples of the Fe_1.95_P_0.8−*x*_Si_0.2_B*_x_* series. Figure 3 illustrates the result for *x* = 0.05. The effective composition of the main phase presents an experimental Fe:(P,Si) ratio of 2.09, reasonably close to the nominal one of 2.05. Small inclusions of a darker phase are observed and their Fe:(P,Si) ratio of 3.11 is reasonably close to that expected for the Fe_3_Si secondary phase. SEM/EDX data therefore provides support to the interpretation of the XRD patterns on the presence of a limited secondary phase close to Fe_3_Si in nature. Elemental mapping for boron shows that it is relatively well distributed within the main phase for *x* ≤ 0.125. Although boron is detected by EDX, its low fluorescence energy compared to that of P, Si, or Fe prevents reliable quantitative determination of its content. The composition of additional secondary phases appearing for *x* ≥ 0.15 could not be resolved. Nevertheless, imaging reveals the presence of extra contrast variations, indicating a multiphase nature of the samples above the solubility limit, in agreement with the XRD results.

**Figure 4:**
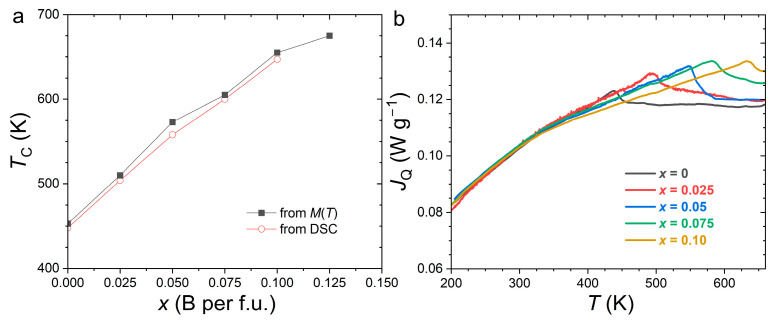
(**a**) Curie temperature as a function of boron content for Fe_1.95_P_0.8−*x*_Si_0.2_B*_x_* compounds determined from the minima of d*M*/d*T* plots on *M*(*T*) curves recorded in µ_0_*H* = 0.05 T and from DSC curves upon heating (*H* = 0). (**b**) DSC heat flow curves upon heating (endothermic).

Figure 4 summarizes the evolution of the Curie temperature (*T*_C_) determined from isofield magnetization (applied field *µ*_0_*H* = 0.05 T) and DSC (*H* = 0) measurements carried out as a function of the temperature. Both datasets are in good agreement and consistently indicate a rapid increase in the Curie temperature with increasing B substitution. The nearly constant discrepancy of ~5 K between the two methods can likely be attributed to small differences in the thermal calibration of the respective instruments as well as to the different criteria used to define *T*_C_ (d*M*/d*T* minimum or right-hand side of DSC peak). The thermal anomalies associated with the magnetic transition remain weak and broad, which is characteristic of a second-order magnetic transition in the Ehrenfest classification. Moreover, no additional thermal anomaly—particularly, no strong DSC signal indicative of a temperature-induced structural transition—was detected within the investigated 200–673 K temperature range. The Fe_3_Si secondary phase is ferromagnetic, with a high Curie temperature (≈805 K) and low magnetic anisotropy; therefore, its presence does not interfere with determining the *T*_C_ of the primary phase.

With increasing B content, the *T*_C_ rises from 453 K in the boron-free compound to 675 K at *x* = 0.125, corresponding to an average rate of increase d*T*_C_/d*x* ≈ +18 K per at.% B. This enhancement of the Curie temperature is comparable to that reported for the ternary Fe_2_(P,B) system (~+15 K per at.% B) [34], and significantly larger than that observed in quaternary (Fe,Co)_2_(P,B) (~+6 K per at.% B) [24] and (Mn,Fe)_2_(P,Si,B) systems (~+10 K per at.% B) [35].

The particularly strong sensitivity of the *T*_C_ to B substitution can be rationalized by the pronounced anisotropic lattice deformation induced by B incorporation, which primarily reduces the *c/a* ratio while preserving the hexagonal Fe_2_P structure. Such a reduction in the interlayer spacing between the 3*f* and 3*g* Fe layers is expected to strengthen the interlayer exchange interactions, thereby stabilizing the ferromagnetic state and leading to a substantial increase in the *T*_C_ [22,32].

**Figure 5:**
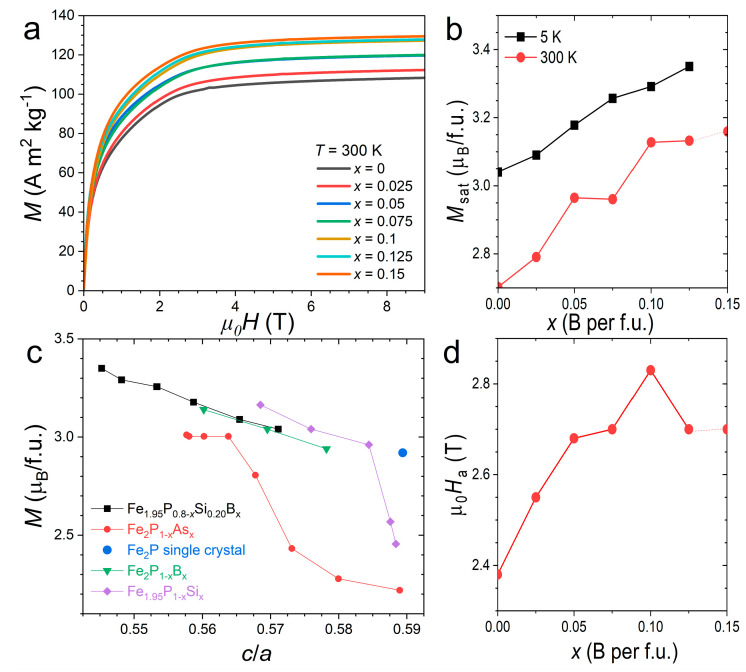
Isothermal magnetization measurements on Fe_1.95_P_0.8−*x*_Si_0.2_B_x_ bulk polycrystalline samples. (**a**) Room temperature magnetization curves; (**b**) saturation magnetization at *µ*_0_*H* = 9 T (recorded at *T* = 5 K and room temperature); (**c**) saturation magnetization presented as a function of the *c/a* lattice parameter ratio [20,28,34,39]; (**d**) room-temperature anisotropy field determined by SPD method.

Figure 5 presents isothermal magnetization measurements performed at *T* = 5 K and 300 K in an applied magnetic field up to *µ*_0_*H* = 9 T. First, a clear increase in saturation magnetization is observed upon B substitution. For the highest B content before reaching the solubility limit (*x* = 0.125), the magnetization reaches 3.36 µ_B_/f.u., which is significantly larger than that of the parent compound Fe_2_P (2.92 µ_B_/f.u. [20]). Considering a theoretical density of 6.60, this corresponds to a maximum polarization of *J* ≈ 1.15 T. When comparing different metalloid substitutions, the Fe_1.95_P_0.8−*x*_Si_0.2_B*_x_* series follows the usual trend in which smaller *c/a* ratios stabilize larger magnetic moments, in particular on the 3*f* site. However, the large dispersion in reported magnetization values, arising from the difficulty of synthesizing pure Fe_2_P and the limited number of experimental studies focusing solely on metalloid substitutions, makes it difficult to assess a universal scaling relationship between magnetization and structural distortion.

We note that these results confirm the first-principle calculations for Fe_2_P_2/3_B_1/3_, which predict that B substitution leads to an increased magnetic moment [40]. However, this observation partly contrasts with results reported for the magnetocaloric (Mn,Fe)_2_(P,Si,B) series, where B substitution was reported to preserve the saturation magnetization [35], and where first-principle calculations do not predict an increase in the magnetic moment [41]. This discrepancy is likely related to differences in structural evolution, particularly in the *c/a* ratio. In the (Mn,Fe)_2_(P,Si,B) series, B substitution significantly decreases the *c/a* ratio in the paramagnetic phase, whereas in the present Fe_1.95_(P,Si,B) series the structure of the ferromagnetic state is primarily affected.

At room temperature, the increase in maximum magnetization at *µ*_0_*H* = 9 T with B is even more noticeable, as it benefits from both the increase in saturation magnetization and the higher Curie temperature. In bulk polycrystalline samples with randomly oriented grains, relatively large magnetic fields are required to saturate the magnetization, which is typical of ferromagnetic materials exhibiting significant magnetocrystalline anisotropy. The anisotropy field (*H*_a_) was estimated using the Single-Point Detection (SPD) method, in which *H*_a_ appears as a minimum on the d^2^*M*/d*H*^2^ curve [42]. An overall increase in *H*_a_ is observed with increasing B content. Interestingly, the highest anisotropy at room temperature is obtained for compositions slightly below the solubility limit, suggesting that the anisotropy at finite temperatures results from a compromise between the enhanced Curie temperature and the reduction in the ground-state anisotropy with increasing B content.

**Figure 6:**
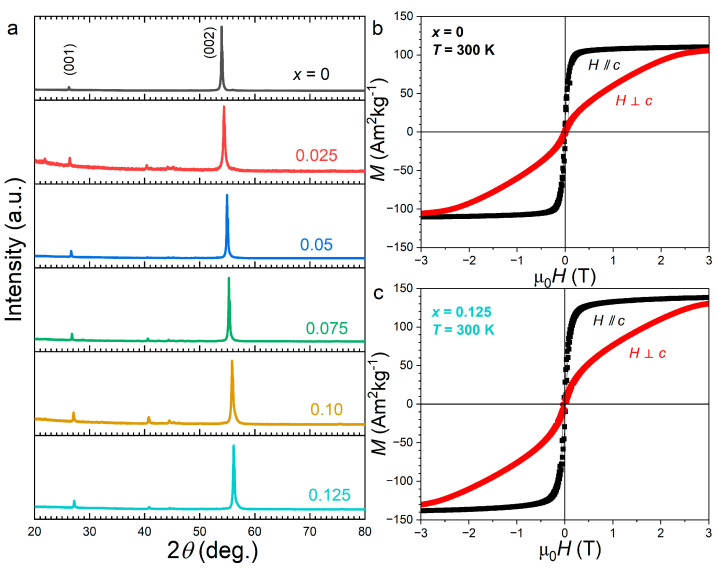
(**a**) Room-temperature XRD pattern of magnetically oriented Fe_1.95_P_0.8−*x*_Si_0.2_B*_x_* powders embedded in epoxy; (**b**) magnetization curves on the magnetically oriented Fe_1.95_P_0.8_Si_0.2_ sample recorded parallel and perpendicular to the orientation field (normalized with respect to magnetic phase fraction only); (**c**) same as panel (**b**) for Fe_1.95_P_0.675_Si_0.2_B_0.125_.

Figure 6 displays room-temperature XRD patterns recorded on magnetically oriented Fe_1.95_P_0.8−*x*_Si_0.2_B*_x_* powders and embedded in epoxy. The diffractograms present a stark contrast compared to the random powders presented in Figure 1, with the most prominent (001) and (002) diffraction peaks aligned along the direction of the magnetic field used for orientation. This indicates that the *c* axis is the easy magnetic direction in this Fe_1.95_P_0.8−*x*_Si_0.2_B*_x_* series, even up to the highest achievable B content before the solubility limit. The clear shift in the (002) peaks to higher angles with B confirms the *c* axis contraction which is responsible for most of the evolution of the magnetic properties.

Magnetization curves were recorded with the magnetic field applied parallel and perpendicular to the orientation direction, confirming significant magnetic anisotropy. Comparison between the boron-free sample and the *x* = 0.125 composition confirms the increase in both the room-temperature saturation magnetization and the anisotropy, with the parallel and perpendicular measurements merging near *µ*_0_*H*_a_ ≈ 2.5 T for x = 0 and 3 T for *x* = 0.125. The pronounced rounding of the perpendicular magnetization curves, however, suggests that the orientation process was only partial and may also include a contribution from the ferromagnetic Fe_3_Si secondary phase, which exhibits low anisotropy. Therefore, further quantitative investigations of magnetocrystalline anisotropy will require the growth of single crystals.

Bulk polycrystalline samples and 36 μm-sized powder particles do not exhibit significant magnetic hysteresis. This observation is consistent with previous single-crystal and polycrystalline studies, which showed that specific preparation methods, such as reducing the particle size to the submicrometric scale, are required to observe hysteresis [25]. This possibility was confirmed by milling a Fe_1.95_P_0.675_Si_0.2_B_0.125_ bulk polycrystalline sample for 5 h into fine particles (details in the Supplementary Materials). The resulting coercive field of about 1 kOe at room temperature is comparable to previous reports [25,27,29]. It remains, however, significantly smaller than the anisotropy field and the low *H*_C_/*H*_a_ ≈ 3.5% ratio suggests that further microstructural engineering will be required to convert the large magnetization, strong anisotropy, and high Curie temperature into effective hard magnetic properties.

## 4. Conclusions

The crystal structure and magnetic properties of quaternary Fe_1.95_P_0.8−*x*_Si_0.2_B*_x_* compounds were systematically investigated. A solubility limit is observed for *x* > 0.125, which is close to that previously reported in ternary Fe_2_(P,B) and quaternary (Fe,Co)_2_(P,B) or quinternary (Mn,Fe)_2_(P,Si,B) compounds. This suggests that the host Fe_2_P-type compound has little influence on the solubility limit and that B incorporation is primarily governed by Hume–Rothery considerations, i.e., the size and valence differences between B and P. Even in already Si-substituted Fe_1.95_P_0.8−*x*_Si_0.2_B*_x_* samples located near the orthorhombic phase boundary, B for P substitutions remain effective in producing a significant reduction in cell volume, which is highly anisotropic with a strongly reduced *c/a* ratio, significantly influencing magnetic properties.

From the perspective of optimizing the intrinsic properties of Fe_2_P-based compounds for permanent magnet applications, B substitution increases the Curie temperature up to 675 K at *x* = 0.125 while preserving the hexagonal structure and the uniaxial easy axis along the *c* direction. At the same time, the saturation magnetization increases, resulting in enhanced magnetization and magnetic anisotropy at room temperature, which highlights the potential of quaternary Fe_1.95_P_0.8−*x*_Si_0.2_B*_x_* compounds as rare-earth-free permanent magnets. Importantly, this substitution strategy enables high Curie temperatures while maintaining the hexagonal Fe_2_P structure without requiring substitutions on the Fe sublattice.

This work also raises new questions that warrant further investigation. While B for P substitutions increase the Curie temperature in both the present Fe_1.95_(P,Si,B) series and the giant magnetocaloric (Mn,Fe)_2_(P,Si,B) materials, only in the former is a significant increase in magnetization observed. This difference calls for dedicated theoretical and experimental investigations. A more accurate determination of the magnetocrystalline anisotropy based on single crystals would also be required, particularly to compare the effects of changes in electron count associated with B for P or Co for Fe substitutions on the anisotropy, and to confirm whether a metalloid-only substitution strategy is indeed the most effective. Finally, achieving hard magnetic properties will require the development of specific microstructures and preparation routes, a task that can be advantageously pursued using the optimized compositions identified in this work.

## Data Availability

The original contributions presented in this study are included in the article. Further inquiries can be directed to the corresponding author.

## Supplementary Materials

The following supporting information can be downloaded at: https://www.mdpi.com/article/10.3390/ma19081579/s1, Figure S1: XRD Rietveld refinements; Figure S2: Magnetic data for a second independent batch of Fe_1.95_P_0.8−*x*_Si_0.2_B*_x_* samples with *x* = 0.05 and 0.10; Figure S3: Magnetic hysteresis of ball milled Fe_1.95_P_0.675_Si_0.2_B_0.125_ powders.
