# Supplementary material for: Structure and Magnetic Properties of Fe1.95P0.8−xSi0.2Bx Compounds: Pushing Beyond the Orthorhombic Limit
Source: Materials (Basel). 2026 Apr 15;19(8):1579. doi: 10.3390/ma19081579 (PMC13117180; doi:10.3390/ma19081579)
Supplement: Supplementary file 1 [file materials-19-01579-s001.zip › materials-4228043-supplementary.pdf]

Supplementary Material for Manuscript « Structure and magnetic properties of $Fe_{1.95}P_{0.8-x}Si_{0.2}B_x$ com-pounds: pushing beyond the orthorhombic limit"

By B. Dorina, L. Bao, B. Axida, B. Wurentuya, Z. Surilemu, F. Guillou and H. Yibole (hyibole@imnu.edu.cn)

Submitted to Materials (MDPI) in March 2026

Supplementary Figure S1 : XRD Rietveld refinements

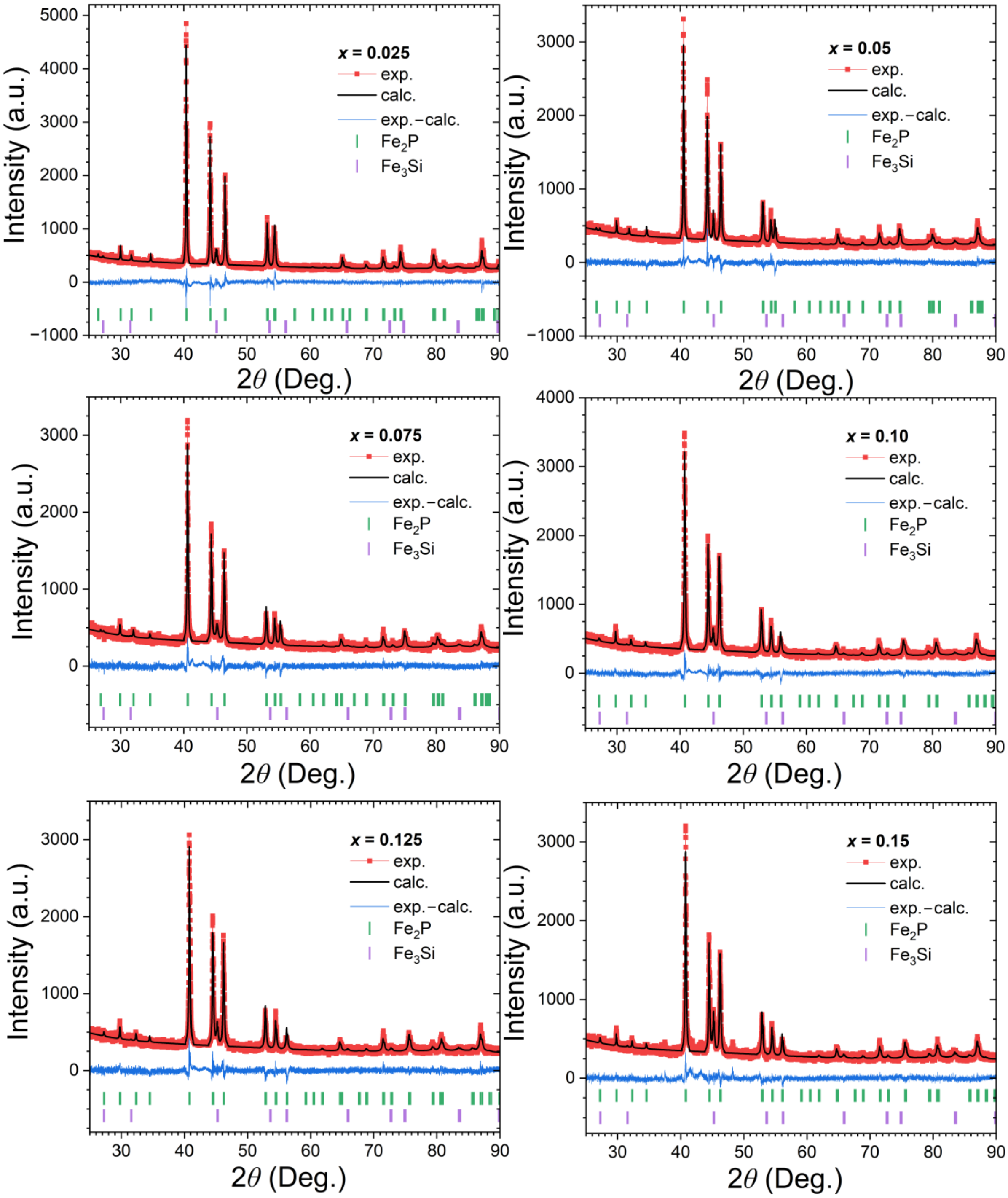

Figure S1: Rietveld refinements of XRD patterns collected at room temperature for $Fe_{1.95}P_{0.8-x}Si_{0.2}B_x$ powders for $0 < x \leq 0.15$. The presence of unindexed peaks for $x = 0.15$ highlights that the solubility limit is reached around this composition.

Supplementary Figure S2: Magnetic data for a second independent batch of $Fe_{1.95}P_{0.8-x}Si_{0.2}B_x$ samples with x = 0.05 and 0.10

The reproducibility of the reported results was checked by synthesizing and characterizing a second batch of samples with *x* = 0.05 and 0.1. Both samples were found to crystallize in the Fe_2P-type hexagonal structure (and presenting traces of Fe_3Si secondary phase) with lattice parameters comparable to those presented in the main text.

Supplementary Figure S2 illustrates their magnetic properties, which are in good agreement with those presented in the main text The maximum magnetization at room temperature under 5 T is 117.8 and 125.7 A m^2 kg^{-1} for *x* = 0.05 and 0.10, respectively, i.e. within 1 and 2% of the values presented in Figure 5. The shape of the M-H curve is also characteristic of the presence of a significant magnetic anisotropy. Similarly the Curie temperatures determined from the minima in d*M*/d*T* differ by approximately 2 K and 4 K for x = 0.05 and 0.10, respectively, compared to those reported in the main text.

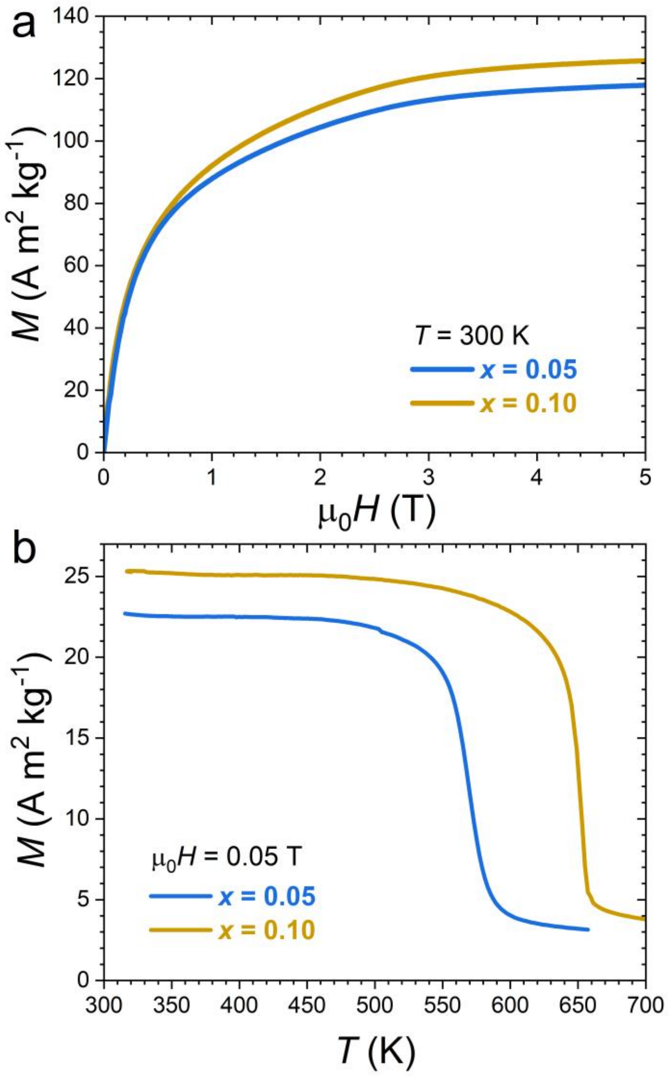

Figure S2: Panel a, Isothermal magnetization measurements at room temperature for an independent batch of $Fe_{1.95}P_{0.8-x}Si_{0.2}B_x$ bulk polycrystalline samples. Panel b, Isofield magnetic measurements upon heating.

Supplementary Figure S3: Magnetic hysteresis of ball milled $Fe_{1.95}P_{0.675}Si_{0.2}B_{0.125}$ powders

The $Fe_{1.95}P_{0.675}Si_{0.2}B_{0.125}$ sample prepared by solid state reaction as described in the main text was subsequently ball milled for 5 h in stainless steel jars using a ball:sample ratio of 6:1. The magnetic properties of the resulting sub-micrometric powders (pressed, not oriented) are presented in Figure S3. One can observe an opening of the hysteresis curve with a coercive field of 1.02 kOe.

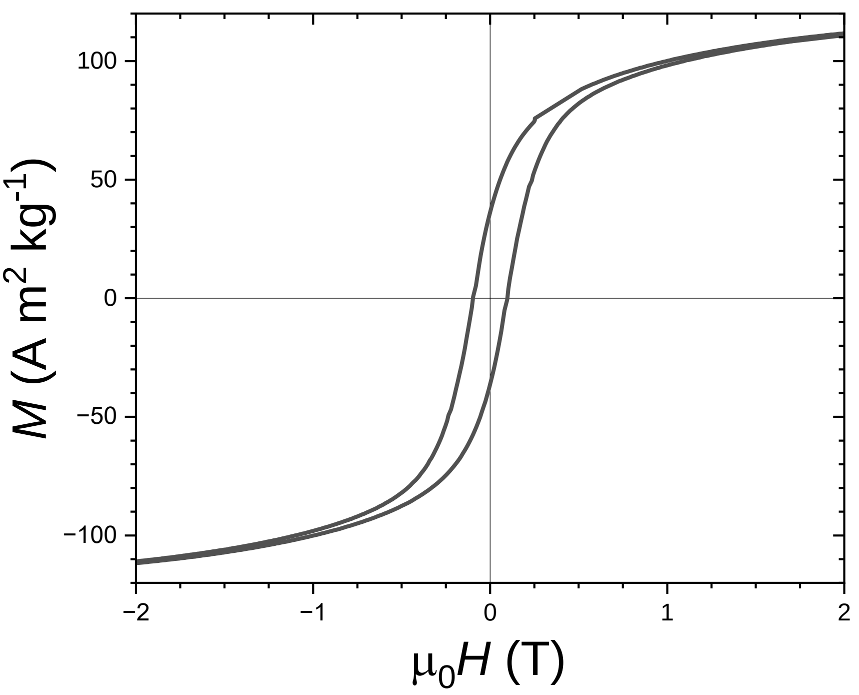

Figure S3 : Magnetic measurements for $Fe_{1.95}P_{0.675}Si_{0.2}B_{0.125}$ sub-micrometric particles.
